# Passive Auditory Stimulation Improves Vision in Hemianopia

**DOI:** 10.1371/journal.pone.0031603

**Published:** 2012-05-29

**Authors:** Jörg Lewald, Martin Tegenthoff, Sören Peters, Markus Hausmann

**Affiliations:** 1 Department of Cognitive Psychology, Faculty of Psychology, Ruhr University Bochum, Bochum, Germany; 2 Research Group Ageing and CNS Alterations, Leibniz Research Centre for Working Environment and Human Factors, Ardeystr, Dortmund, Germany; 3 Department of Neurology, BG-Kliniken Bergmannsheil, Ruhr University Bochum, Bochum, Germany; 4 Department of Radiology, BG-Kliniken Bergmannsheil, Ruhr University Bochum, Bochum, Germany; 5 Department of Psychology, University of Durham, Durham, United Kingdom; University of Regensburg, Germany

## Abstract

Techniques employed in rehabilitation of visual field disorders such as hemianopia are usually based on either visual or audio-visual stimulation and patients have to perform a training task. Here we present results from a completely different, novel approach that was based on passive unimodal auditory stimulation. Ten patients with either left or right-sided pure hemianopia (without neglect) received one hour of unilateral passive auditory stimulation on either their anopic or their intact side by application of repetitive trains of sound pulses emitted simultaneously via two loudspeakers. Immediately before and after passive auditory stimulation as well as after a period of recovery, patients completed a simple visual task requiring detection of light flashes presented along the horizontal plane in total darkness. The results showed that one-time passive auditory stimulation on the side of the blind, but not of the intact, hemifield of patients with hemianopia induced an improvement in visual detections by almost 100% within 30 min after passive auditory stimulation. This enhancement in performance was reversible and was reduced to baseline 1.5 h later. A non-significant trend of a shift of the visual field border toward the blind hemifield was obtained after passive auditory stimulation. These results are compatible with the view that passive auditory stimulation elicited some activation of the residual visual pathways, which are known to be multisensory and may also be sensitive to unimodal auditory stimuli as were used here.

**Trial Registration:**

DRKS00003577

## Introduction

In the mammalian brain, auditory and visual systems are closely interconnected. Single-neuron recordings in non-human species have demonstrated auditory-visual bisensory responses as well as effects of cross-modal integration in a multitude of subcortical and cortical regions. These comprise the inferior and superior colliculi, the thalamus and frontal, temporal, insular, parietal, and occipital cortices which include the presumptive unimodal sensory areas. Moreover, neuroimaging studies have indicated potential correlates of bisensory phenomena in human cortex [1], [2]. These interconnections between the senses may form the structural basis for the remarkable capacity of spatial cross-modal plasticity which can occur in a surprisingly short time scale (e.g., [3], [4]).

Currently, the issue of cross-modal spatial plasticity attracts growing attention with respect to patients with homonymous hemianopia (HA). This is a visual field defect, characterized by a loss of vision in one hemifield. It is caused by unilateral brain lesions located contralaterally to the anopic side in postchiasmatic optic tract, lateral geniculate nucleus, optic radiation or (in the majority of cases) in the occipital lobe, while leaving intact the superior colliculus (SC). The functional integrity of the SC in HA may result in the retention of some residual visual functions (at a relatively low unconscious level without acknowledged awareness) in their anopic hemifield which has been referred to as blindsight [5]–[10]. These residual abilities in HA have usually been proposed to rely on “residual” visual pathways that are independent of the damaged geniculostriate pathway to the primary occipital area (V1). In particular, visual information may be conveyed from the eye directly to the SC, from there to the pulvinar nucleus of the thalamus which, in turn, projects not only to parietal but also onto temporal (including primary auditory) cortices [6], [9], [11]–[13]. Alternatively, in cases where the lateral geniculate nucleus is left intact, projections from this structure to extrastriate cortex may enable V1-independent processing of visual information [14]. However, residual visual functions can also be based on surviving visual fibres of the geniculostriate pathway [15]–[17]. Thus, residual visual abilities in the anopic hemifield of HA patients can, in principle, rely on two types of residual fibers: *(1)* intact fibers of the extrastriate visual pathways and *(2)* surviving fibers in the partially damaged primary visual system.

It is important to note that almost all brain regions involved in both types of these “residual” pathways are multimodal structures, which are not only involved in processing of auditory and visual information, but also in the allocation of attention across sensory modalities. The SC contains superimposed maps of visual and auditory spaces, in particular topographically-aligned eye-centred bisensory representations of contralateral hemispace, located in the deep and intermediate layers [18], [19]. This structure is also known to be crucially involved in the control of both overt and covert spatial attention [20], [21]. With respect to the pulvinar nucleus, Cappe et al. [22] recently suggested that it receives multisensory information from the SC and, referring to the hypothesis of Crick and Koch [23], loops between cortex and pulvinar may be part of mechanisms involved in multisensory integration observed in unisensory cortical areas. Neurons in the posterior parietal cortex, that is known to receive inputs from the pulvinar [24] and to have direct interconnections with ipsi- and contralateral SC [25], have been shown to support the integration of auditory and visual space [26], [27] as well as the control of auditory and visual spatial attention [28], [29]. Auditory-visual interaction has also been demonstrated in single cells in the cortex of the superior temporal sulcus [30]. Finally, there is sufficient evidence from human and animal research that even the primary visual system, namely the lateral geniculate nucleus [31], [32] and the primary visual cortex [33]–[39], exhibits properties of auditory-visual cross-modal interaction.

In accordance with these findings, the relevance of auditory-visual bisensory integration of information has been demonstrated in patients with HA. It has been found that a sound, spatially and temporally coincident to a visual stimulus, can improve visual perception in the blind hemifield of hemianopic patients [40]. In addition, auditory localization performance in the blind hemifield was markedly enhanced when a visual stimulus was coincident with the acoustic target in both space and time [41]. Adaptation by spatially coincident repetitive auditory-visual stimulation induced significant improvement in auditory localization after exposure [42]. Most importantly, systematic training with auditory spatial stimuli presented in spatio-temporal alignment with visual stimuli, was shown to induce long-lasting visual improvement in visual search in the anopic hemifield [43].

As with previous approaches, the present study started from the largely accepted basic hypothesis that sensory input from an intact modality (audition) can improve processing of information by spared structures of a damaged sensory system (vision). Activation of the colliculo-pulvinar-extrastriate pathway and/or surviving parts of the primary pathway in HA may induce an improvement of the related residual visual abilities in the blind field, either by more effective sensory processing of unimodal visual information within the “residual” pathway, or by an increase of spatial attentional functions. We made the assumption that auditory-visual bimodal neurons not only respond to stimulus combinations from different modalities, but also can show suprathreshold responses to unimodal stimuli, thus providing a substrate for signalling in two *separate* modalities, despite their potential for *integrating* information from different modalities (cf. [44]). Previous bimodal approaches to improve blind-field vision in HA (e.g., [45]) have focussed on the latter issue, correctly arguing that combining auditory and visual stimuli may be more beneficial than unimodal stimuli in this respect, given the known mechanisms of multisensory interaction, in particular those of multisensory enhancement observed in SC neurons (for review, see [1]). However, while such multisensory *integrative* properties may be present in a relatively small portion of cells, it is known that the vast majority of neurons in SC and in posterior parietal cortex show multisensory *sensitivity*, that is, most neurons that are responsive to visual stimuli respond equally well or even more strongly to unimodal auditory stimuli (e.g., [18], [27]). Thus, as separate modalities are processed by the same neurons and the same synapses, it is reasonable to suggest that activation of these multimodal circuits by unimodal auditory stimuli could induce facilitating effects of unimodal visual information within these bimodal pathways. We therefore used unimodal auditory stimuli for activation of the multisensory “residual” pathways to test whether this stimulus type is suited to induce improving effects on blind-field vision in HA.

While earlier approaches used training procedures involving the execution of specific tasks, we employed a protocol of passive auditory stimulation (PAS). This protocol closely follows the idea that synchronous neural activity, necessary to drive plastic changes, is evoked by repetitive sensory stimulation without requiring any active task from the patient [45]. Such task-free, passive stimulation protocols, also referred to as *coactivation* or *unattended activation-based learning*, have been shown in several previous studies to improve tactile and sensorimotor performance in healthy human subjects as well as in subacute and chronic stroke patients [46]–[49]. We hypothesised that PAS on the anopic side of HA patients may induce short-term cross-modal effects, resulting in an improvement of vision immediately after PAS. Since the SC contains a map of the contralateral half of the auditory space (for review, see [1]), we assumed that hemispatial PAS on the anopic side (thus selectively activating the residual pathway in the damaged cerebral hemisphere) would be more effective than PAS on the side of the intact hemifield.

## Materials and Methods

### Ethics Statement

This study conformed to the Code of Ethics of the World Medical Association (Declaration of Helsinki), printed in the British Medical Journal (18 July 1964). All patients gave their written informed consent to participate in this study, which was specifically approved by the Ethical Committee of the Medical Faculty of the Ruhr University Bochum.

### Patients

**Figure 1:**
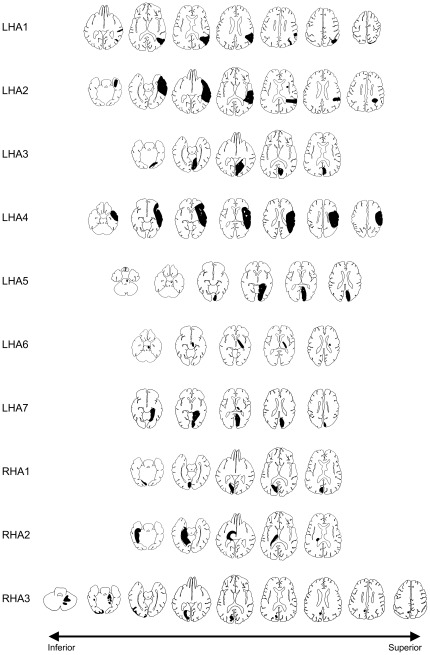
Lesion sites. Series of schematic brain slices along the superior-inferior direction for each of the ten patients are depicted using standardized templates from Damasio and Damasio [84], with black areas indicating the lesioned sites. More inferior templates are to left, more superior templates to the right. Templates are in neurological orientation, i.e., the left side of the template refers to the left side of the brain.

Ten patients with brain lesions participated in this study. All of them had received the diagnosis of persistent homonymous hemianopia (HA) confined to one hemifield, as confirmed by visual perimetry. HA was left-sided (LHA) in seven patients (LHA1–LHA7) and right-sided (RHA) in three patients (RHA1–RHA3). Age, sex, visual field defects and lesion sites are reported in Table 1 (detailed information on lesion sites is given in Fig. 1). All patients were congenitally right-handed, as assessed by a German adaptation of Coren's [50] inventory [51], with a criterion of an individual score of ≥2 (range from −4 to 4) in the hand section of this questionnaire. However, hemiparesis prevented two patients (LHA6, RHA2) from use of their contralesional hand, and two other patients (LHA4, LHA7) showed mild impairment with use of the contralesional hand due to hemiparesis. Two further patients (one LHA and one RHA patient) were also tested, but were excluded from the study. One of the excluded patients already showed excessive variation in the position of the VFB (maximum difference >20 degrees) between baseline measurements, and the other patient was unable to follow adequately the instruction to fixate on the central fixation target during experimental blocks (see below). All patients were naïve with respect to the purpose of the experiment.

**Table 1:** Summary of clinical data and visual field defects of patients with hemianopia.

Patient	Age	Sex	Side of HA	VF border	Time since onset	Ethiology	Lesion site
LHA1	39	F	Left	−1.7°	10 months	AVM	Right temporo-parieto-occipital
LHA2	61	M	Left	−3.8°	7 years	ICH	Right temporal
LHA3	64	M	Left	−2.3°	9 months	CI	Right temporo-occipital
LHA4	23	F	Left	−5.1°	44 months	CI, ICH	Right temporo-parieto-frontal
LHA5	65	M	Left	−3.4°	6 years	CI	Right occipital
LHA6	43	F	Left	−0.1°	43 months	CI	Right temporal
LHA7	40	M	Left	−1.8°	6 years	CI	Right occipital
RHA1	44	M	Right	+13.0°	5 months	CI	Left temporo-parieto-occipital
RHA2	48	M	Right	+0.7°	34 months	CI	Left temporo-occipital
RHA3	37	F	Right	+3.0°	8 months	CI	Left parieto-occipital, right occipital

VF borders are means across pre-PAS blocks in all three experimental conditions. Negative VF borders are to the left, positive to the right.

*Abbreviations* AVM, cerebral arteriovenous malformation; CI, cerebral ischemia; ICH, intracerebral hemorrhage; HA, hemianopia; PAS, passive auditory stimulation; VF, visual field.

All HA patients had circumscribed brain lesions as a result of ischemic stroke or haemorrhage, demonstrated by magnetic resonance imaging (MRI) or computed tomography (CT). In all patients, lesions were unilateral (i.e., on the side contralateral to the anopic hemifield), with the exception of patient RHA3 who showed some minor involvement of right-hemispheric regions in addition to the predominant left-hemispheric lesion (see Fig. 1).

To test whether HA patients suffer from spatial neglect a neglect-test battery [52] was applied, which consisted of the following tests: *(a)* Letter Cancellation task [53], which requires the patient to cancel all target letters ‘A’ (30 on the left and 30 on the right side) distributed amid distractors on a horizontally oriented standard page (DIN A4). Patients are classified as suffering from spatial neglect when they omit at least five targets on the left. *(b)* Bells Test [54], which consists of seven columns each containing five targets (bells) and 40 distractors. Three of the seven columns ( = 15 targets) are located on the left and three columns ( = 15 targets) are located on the right side of a horizontally oriented standard page. When patients omit more than five left sided targets, they are classified as suffering from spatial neglect. *(c)* Baking Tray Test [55], which requires the patient to place 16 identical items as evenly as possible on a blank standard page (8 on the left, and 8 on the right side). Any distribution more skewed than seven items on the left side and nine items on the right side is considered as a sign of spatial neglect. *(d)* Copying task [52], [56], in which patients are asked to copy a complex multi-object scene consisting of four figures on a standard page (two on the left, and two on the right side). Omission of one left-sided feature of each figure is scored as 1, and omission of each whole figure is scored as 2, resulting in a maximum score of 8. A score higher than 1 (i.e. >12.5% omissions) is considered as a sign of spatial neglect. None of the HA patients exceeded the limit values in at least two of these four tests, which has been regarded as the criterion for presence of spatial neglect [57].

In addition, we applied a line-bisection task which comprised 17 horizontal black lines of 1 mm width on a horizontally-oriented white standard page. The lines ranged from 100 to 260 mm long, in steps of 20 mm. The mean length was 183.5 mm. Patients were asked to bisect all lines into two parts of equal length by marking the subjective midpoint of each line with a fine pencil (for details, see e.g. [58], [59]). Neglect patients typically show a large bisection bias towards the right. Unlike that, the HA patients showed a significant mean bisection bias of 4.05% (SE 1.70, range from −2.92% to 11.32%; *t*[9] = 2.38, *p* = 0.041) toward the side of the anopic hemifield (LHA: mean leftward bias 6.01%, SE 1.94; RHA: mean rightward bias 0.53%, SE 1.47). This conforms with previous findings of a contralesional bias in patients with HA (e.g., [58]–[60]).

**Figure 2:**
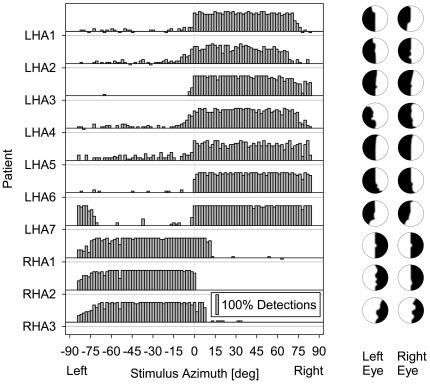
Visual field defects of patients with left (LHA1–7) and right hemianopia (RHA1–3). The left panel shows the azimuthal dimensions of the binocular visual fields, as were measured without PAS (mean of pre-PAS blocks 1 and 2 across all three sessions). For each patient, the percentage of correct detections (gray bars) of visual stimuli is plotted as a function of the stimulus azimuth (steps of 2°; negative azimuths, left hemifield; positive values, right hemifield). The right panel shows reconstructions of the monocular visual fields based on static perimetry (black areas: anopic regions; white areas: intact regions).

Prior to experimentation, the presence of homonymous HA was confirmed by visual static perimetry in all patients included in this study (see plots in Fig. 2). In addition, the azimuthal dimensions of the visual field, and in particular the position of the binocular VFB (see Table 1) was derived from the baseline measurements in each experimental session, using visual stimulation by the experimental apparatus (see below). Across all patients, the baseline VFB was only slightly shifted toward the side of the anopic field (mean 3.48°, SE 1.16°). One of the patients (LHA7) showed incomplete left HA, with a small peripheral area of vision lying to the left of the anopic field.

All patients were tested for general hearing loss. For this purpose, white-noise bursts with a duration of 1 s were presented monaurally via headphones (K271, AKG Acoustics, Vienna, Austria) at various sound-pressure levels (SPLs, range 10–80 dB re 20 µPa, steps of 10 dB; onset/offset time 50 ms), and patients pressed a button as soon as they heard a sound. In this test, HA patients did not show any superiority of the ear on the side of the intact (contralateral) or the anopic (ipsilateral) hemifield (*t*[9] = 0.00, *p* = 1.00).

Prior to these experiments, all HA patients had already participated in studies in which they had been tested for spatial hearing abilities [61], [62]. In these previous studies, patients showed statistically significant deficits in accuracy and precision of sound localization compared with healthy controls. However, in absolute terms, impairments were very slight, such that the patient's general ability to localize a sound could be considered as quasi-normal (for details, see [61], [62]). Each patient completed the first session of the present experiment within 2 to 11 months after these investigations.

### Apparatus and stimuli

The experiments took place in a sound-proof and anechoic room (5.4×4.4×2.1 m^3^), which was insulated by 40 cm (height)×40 cm (depth)×15 cm (width at base) fiberglass wedges on each of the six sides. A suspended mat of steel wires served as floor. The ambient background noise level was below 20 dB(A) SPL.

**Figure 3:**
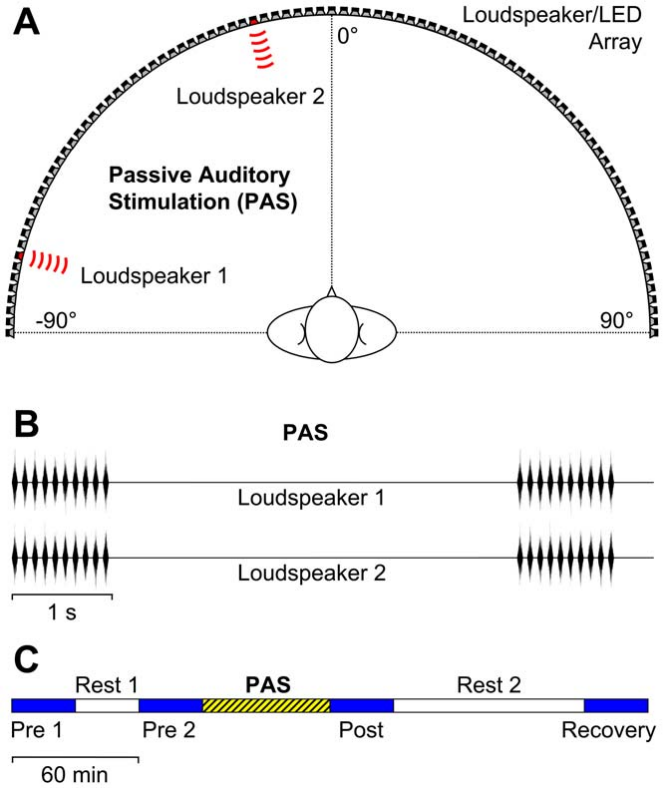
Experimental procedure for passive auditory stimulation (PAS). (**A**) Sounds were delivered simultaneously from two loudspeakers which were both located on the same side, either to the left or right from the patient's median plane. (**B**) During the PAS period, trains of ten 60-ms noise bursts were presented at a rate of 0.2 s^−1^. (**C**) Prior to the PAS, baseline data were obtained in two pre-PAS blocks (Pre 1, Pre 2), with an intermittent rest of 30 min (Rest 1). Immediately after the end of the PAS followed the post-PAS block (Post), and then the patient was allowed to rest (Rest 2). The final block (Recovery) was started 1.5 h after the end of the post-PAS block.

The patient sat on a comfortable chair with their head fixed by a custom-made framework with stabilizing rests for the chin, forehead, and occiput (see [63]). In front of the patient, at a constant distance of 1.5 m from the centre of the head, 91 broad-band loudspeakers (5×9 cm^2^, Visaton SC 5.9, Visaton, Haan, Germany) were mounted in the patient's horizontal plane. The azimuth of the loudspeakers ranged from −90° (left) to 90° (right), in steps of 2°, with the centre loudspeaker at 0°. However, only four of the loudspeakers were used in these experiments (see Fig. 3A): two loudspeakers at −76° and −14° to the left (for left-sided PAS); and two loudspeakers at 14° and 76° to the right (for right-sided PAS).

The PAS protocol used here has been originally developed by J. Lewald, S. Getzmann, and H. R. Dinse (unpublished; [64]). The PAS protocol was adopted from the previous literature on unattended activation-based learning in the tactile modality. We used a high-frequency stimulation protocol (10 Hz) for PAS, as has been shown to induce improvement of sensory performance in these previous studies. It has been suggested that this type of sensory stimulation may evoke processes of long-term potentiation (LTP) of synaptic transmission, thus resulting in activity-dependent strengthening of synaptic connections (e.g., [45]). The acoustic stimulus used consisted of band-pass-filtered frozen noise (lower cutoff frequency 2 kHz; upper cutoff frequency 11 kHz). The cutoff frequencies were chosen on the basis of pilot experiments, in order to maximize the spatial separability, as stimuli were presented simultaneously from two locations. Stimuli were trains of ten sound bursts (Fig. 3B). Each stimulus train had an overall duration of 960 ms; single sound bursts had a duration of 60 ms with triangular envelope (onset/offset time 30 ms) and were presented at a rate of 10 s^−1^. The stimulus trains were presented at a rate of one per 5 s (interstimulus interval 4.04 s). PAS stimuli were always emitted simultaneously from two loudspeakers located 14° and 76° on the same side. Analogously to the previous tactile studies, we presented two stimulus locations simultaneously in order to activate large parts of the multisensory map of contralateral hemispace that is present in SC (for review, see [1]). In order to prevent fusion of the two sound sources into one unified percept, we used incoherent noise, i.e. waveforms delivered by the two loudspeaker channels were independent. Under these conditions, normal subjects typically hear two spatially disparate sounds, or at least a sound image extending over a relatively broad range in azimuth within the stimulated hemifield. Sound stimuli were generated digitally using CoolEdit 2000 (Syntrillium Software Co., Phoenix, AZ, USA) and converted to analog form via a computer-controlled external soundcard (Sound Blaster Audigy 2 NX, Creative Labs, Singapore) at a sampling rate of 96 kHz. Sound stimuli were presented at a sound-pressure level of 60 dB(A).

For visual stimulation, at the lower edge of the chassis of each loudspeaker a white light-emitting diode (LED) was mounted in a central position, thus resulting in an array of 91 LEDs over 180° azimuth centered to the patient's head (Fig. 3A). Each of these LEDs (diameter 10 mm; luminance about 700 cd/m^2^) was mounted in a small housing impermeable to light, with a central circular aperture of 2 mm diameter immediately in front of the LED (resulting in a luminous intensity of about 0.003 mcd). As the aperture resulted in a narrow viewing angle of the LED and the optical axis of the LED was exactly oriented toward the patient's head, straylight was almost completely prevented. Moreover, the experimental setup in front of the patient including loudspeakers and LED housings were matt black, and any specular light-reflecting surfaces did not exist in the experimental room.

Visual stimuli consisted of single light flashes with rectangular envelope (duration 50 ms). In addition, one dim red LED (diameter 3 mm; luminance about 35 cd/m^2^) served as a fixation target. The fixation LED, that was permanently on, was mounted immediately below the central loudspeaker (0° azimuth). In order to control for accurate fixation, eye position was monitored online by the experimenter via an infrared video camera. The video camera was mounted on a long-focus lens and was focussed on the patient's right eye. The fixation LED emitted light of wavelengths from the visible (red) down to the infrared range, and thus also served as the light source for the infrared video camera.

The timing of the stimuli and the recording of the patients' responses were controlled by custom-written software. Reaction times (RTs) were measured by a high-resolution timer interface connected with an external response button. All experimental blocks were conducted in total darkness, except the visual stimuli and the fixation LED. During the PAS period, the experimental room was dimly illuminated (background luminance <10 cd/m^2^) in order to counteract drowsiness of the patients. The room was also illuminated during pauses between experimental blocks and patients had their eyes open prior to the beginning of each block, in order to keep constant the level of pre-adapting luminance. Thus, conditions of dark adaptation were constant for each block (see below).

### Procedure

#### Conditions of PAS

The experiment consisted of three sessions, conducted on different days, with intervals of several weeks to months beween sessions. Patients completed all sessions within 2 to 7 months. Sessions were conducted following a fixed sequence, each session with a different condition of PAS. PAS was applied to the anopic side in Session 1 and to the intact side in Session 2. No acoustic stimulation was presented in the final Session 3, which was used as the control condition (see below).

#### Course of the experimental session

Each session was subdivided into four identical experimental blocks in which patients performed the visual detection task (pre-PAS 1; pre-PAS 2; post-PAS; recovery). Prior to the beginning of the session, patients were familiarized with the experimental set-up and a minimum of 20 practice trials was conducted. After pre-PAS block 1 was completed, the patient was allowed to rest, and pre-PAS block 2 was started 1 h after the beginning of pre-PAS block 1. The PAS period lay between pre-PAS-2 and post-PAS blocks (Fig. 3C). After the end of the PAS period, the post-PAS block was started without a break. After this block was completed, the patient was allowed to rest for about 1.5 h. Finally, the recovery block started 2 h after the end of the PAS period.

#### Visual detection task

In each trial, a white light flash was presented in total darkness. Trials were not announced. The timing of the trial onset was controlled by the computer. The location of the flash changed between trials following a fixed quasi-random order over a range from −90° on the left to 90° on the right, in steps of 2°. Patients were instructed to fixate the central red LED, and to press a response button as soon as a white light flash appeared. Patients were explicitly encouraged to guess and to also respond when they had merely a vague impression that any event appeared in their blind field. The next stimulus followed after a quasi-randomly varied time interval (balanced across trials) between 1 s and 3 s (steps of 0.5 s) after the patients' response ( = trial onset), such that patients were not able to predict the time of stimulus presentation. Results of the first 10 min of the block were discarded, as this period was considered to be necessary for sufficient dark adaptation and to give patients adequate practice with the task. Data were collected in a total of 273 trials, in which each of the 91 stimulus positions was presented three times. The overall duration of each block, including the discarded trials, was about 30 min.

#### PAS

Immediately following the end of pre-PAS block 2, the PAS was begun. The patient was seated with the head fixed, as in the experimental blocks. No specific instruction was given. A total of 720 acoustic stimulus pairs, always emitted simultaneously from the two loudspeakers on the same side, were presented over a period of 1 h. In the control session, no acoustic stimuli were presented during this period (i.e., silence). Apart from that, experimental conditions were as in sessions with PAS.

### Data analysis

**Figure 4:**
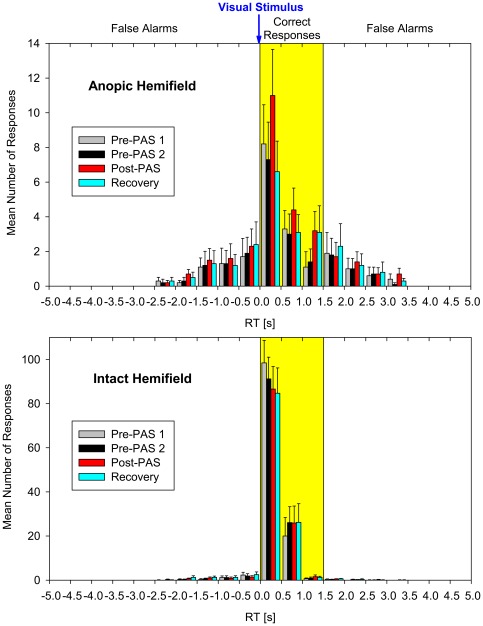
Frequency distribution of reaction times (RTs). RTs obtained in anopic (upper plot) and intact hemifield (lower plot) are shown for the four experimental blocks of the session with PAS on the side of the anopic hemifield. Each bar shows the number of responses to light flashes recorded in a time interval of 0.5 s (mean values across all patients; error bars, standard error; 0 s = stimulus offset). The yellow area indicates the range of RTs within that responses were considered to be correct (1.5 s after stimulus offset). The remaining responses outside this time window were considered to be false alarms.

Responses to light flashes were considered to be correct if the RT was within 1.5 s (see Fig. 4). Data were corrected for false alarms, that is, reactions not controlled by the reaction stimulus. For this purpose, all responses in one to two time intervals (overall duration 1.5 s) between 1 s and 4.5 s after trial onset that were not within the adjacent “correct” time interval of 1.5 s after stimulus offset, were considered as false alarms. For example, when the flash was presented 3 s after trial onset, the 1.5-s interval from 1.55 s after trial onset to stimulus offset was used for estimating false alarms; or when the flash was presented 1.5 s after trial onset, the sum of the periods from 0.5 s before stimulus offset and from 1.5 s to 2.5 s after stimulus offset was used for this purpose. For each experimental block, the mean rate of false alarms was subtracted from the patient's rate of correct responses prior to further data analysis. Across all patients the mean rate of false alarms was 3.23% (SE 1.13). The mean rates of correct detections adjusted for false alarms were 5.84% (SE 1.58) in the anopic hemifield and 81.87% (SE 3.89) in the intact hemifield.

**Figure 5:**
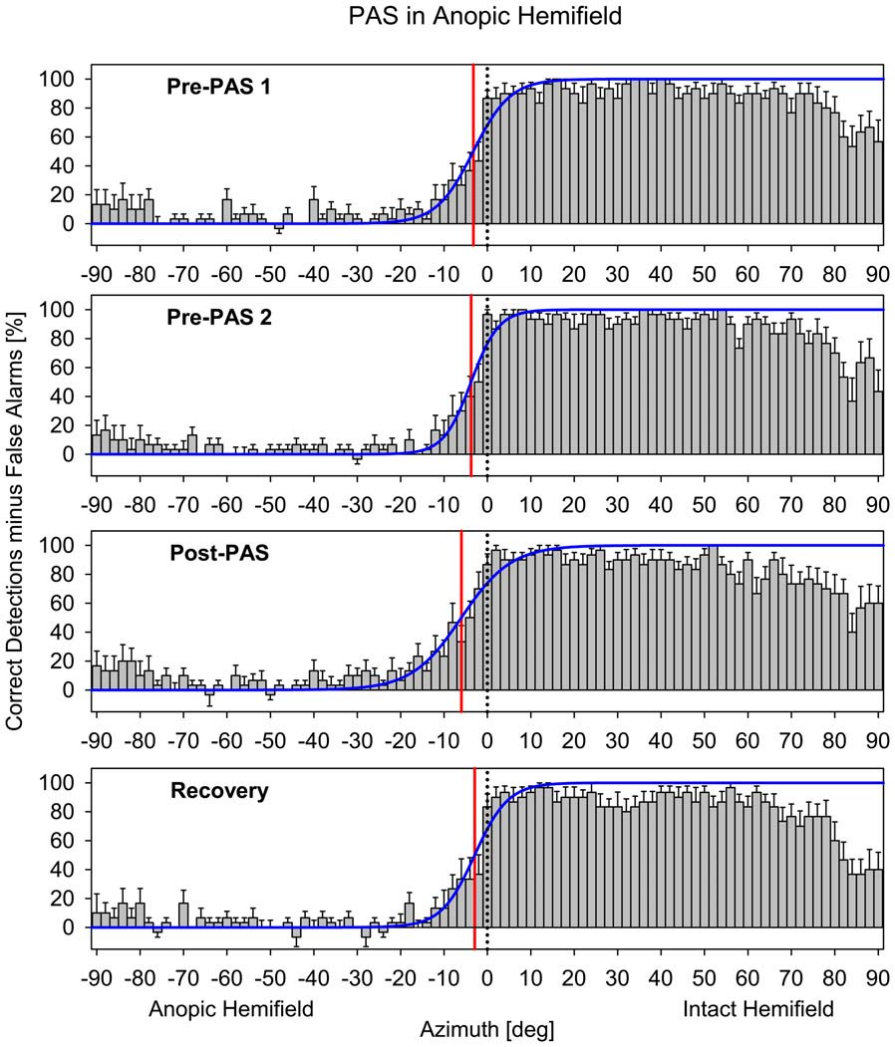
Mean percentage of correct detections minus false alarms, plotted as a function of visual stimulus location, for the four experimental blocks of the session with PAS on the side of the anopic hemifield. Bars indicate mean values across all patients (error bars, standard error). Data for the central range (±46°) were fitted to a sigmoid equation (solid curve, extrapolated to ±90°), and the 50 percent point of the curve was defined as the position of the VFB (vertical solid line). In the post-PAS block, the VFB was shifted by few degrees toward the side of the anopic hemifield compared with the other blocks. Note that the negative percentages at some positions were the result of more false alarms than correct detections. Negative azimuths, anopic hemifield; positive azimuths, intact hemifield; vertical dotted line, median plane.

For statistical comparisons, data of LHA and RHA patients were classified according to whether they had been obtained within the patient's anopic or intact hemifield and were pooled. The resulting percentages of correct responses (minus false alarms) were plotted as a function of stimulus azimuth for each experimental block (Fig. 5). Two different analyses were performed on the basis of these data sets.

The first analysis concentrated on changes in the rate of correct detections depending on the sequence of the experimental blocks. Only detections in anopic hemifield were analysed in detail. We refrained from corresponding analyses in intact hemifield because of the inevitable presence of a ceiling effect (with nearly 100% correct detections in the more central parts of the visual field; see Fig. 5).

In the second analysis, the VFB depending on the sequence of the experimental blocks was derived from the same original data sets. For computation of the VFB, the number of correct responses (minus false alarms) was plotted as a function of stimulus azimuth (*θ*) within the range of −46° on the left to 46° on the right (in order to exclude potential effects of peripheral vision), and fitted to the sigmoid equation:
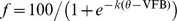
where *f* is the frequency of responses, given as percentage; VFB is that *θ* where *f* is 50%; k is the slope of the function at 50%; *e* the base of the natural logarithm. The mean coefficient of determination (*R* ^2^) of the fit was 0.91 (range from 0.27 to 1.00; *p*≤0.0009 in each case), indicating analyzable boundary of the visual field for all patients. The visual-field border determinations did not differentiate between areas of relative or absolute defect. In both analyses (correct detections, VFB) the mean of the data obtained in blocks 1 and 2 was used as the pre-PAS baseline for each individual patient. In neither case, statistical comparisons of results obtained in blocks 1 and 2 revealed any significant difference (paired *t*-tests; *t*[9]≤0.60, *p*≥0.56).

For statistical analyses, two-factor repeated-measures analyses of variance (ANOVAs) were conducted to compare performances of patients at three measurement points in time (pre-PAS; post-PAS; recovery) and for three conditions of PAS (anopic hemifield; intact hemifield; control condition). In subsequent stages of analysis, one-factor ANOVAs were used to reveal differences between measurement points in time within one session and between conditions for each measurement point in time. For all computations, the Mauchly test of sphericity was checked, and the Greenhouse-Geisser correction was performed when appropriate. The *α*-level was adjusted for multiple testing (Bonferroni). In particular, *α*-adjustments accounted for the two independent analyses (correct detections, VFB) performed on the basis of the same set of data.

## Results

### Effect of PAS on visual detections

**Figure 6:**
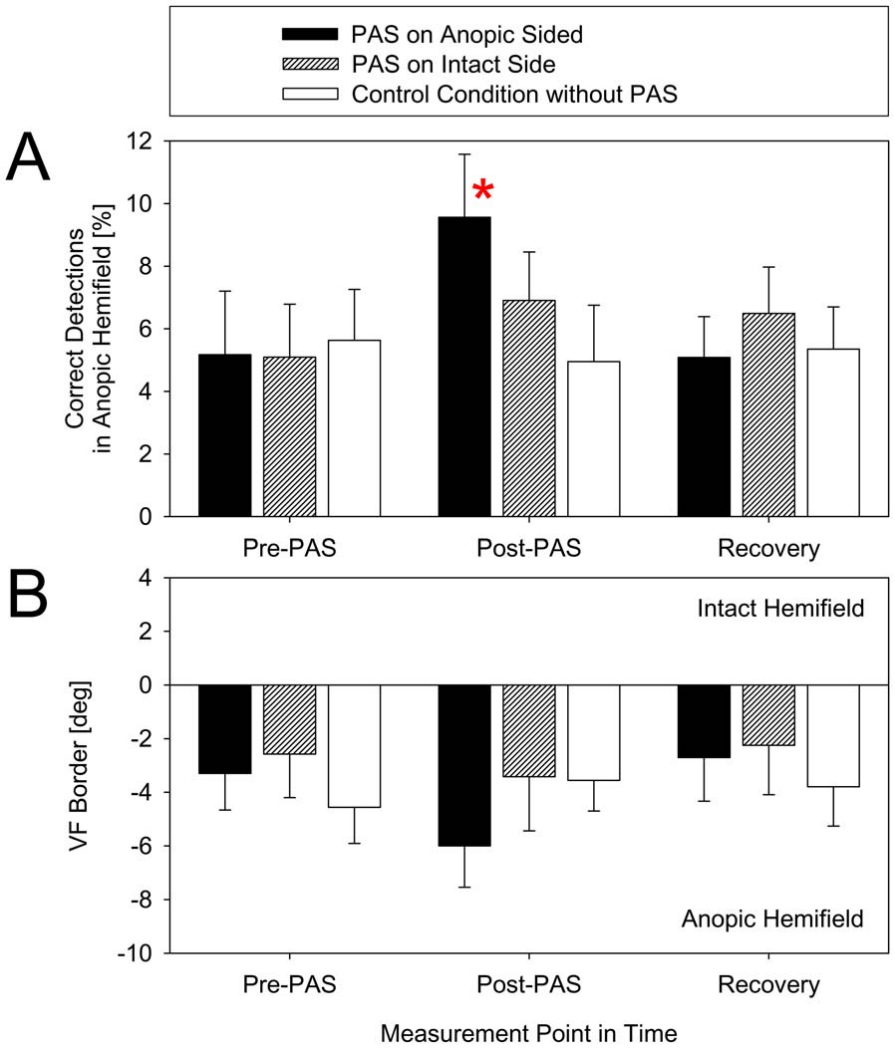
Effects of PAS. Rate of correct detections in the anopic hemifield (**A**) and VFB (**B**) measured prior to PAS (pre-PAS, mean of blocks 1 and 2) are compared with data obtained immediately after PAS (post-PAS) and after a period of recovery in three experimental conditions (PAS on the anopic side; PAS on the intact side; control condition without PAS). Plots show mean values across all patients (error bars, standard error). Statistically significant differences (asterisk; A) were found only for the rate of correct detections with the measurement after PAS on the side of the anopic hemifield. This value differed from both pre-PAS and recovery data of the same session, and from the related measurement point in time of the control condition.

**Figure 7:**
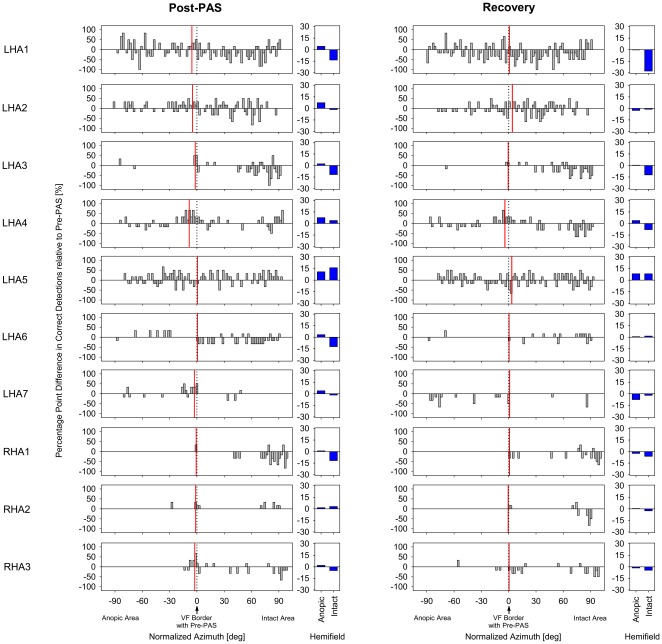
Normalized percentage of correct detections for each individual subject in the post-PAS and recovery conditions of the session with PAS on the side of the anopic hemifield. Gray bars show the difference between percentage values obtained in the respective block and the mean percentage obtained in pre-PAS blocks 1 and 2. The azimuth of visual stimulus location (steps of 2°) is normalized for each subject such that zero (vertical black dotted line) is the mean visual field (VF) border measured in the pre-PAS blocks 1 and 2. Thus, positive gray bars indicate improved detections, and negative positions of the VF border (vertical red solid line) indicate a shift toward the anopic field with reference to pre-PAS blocks. Panels with blue bars show mean changes with reference to pre-PAS blocks across total anopic or intact hemifields (non-normalized azimuth). Note that in their anopic hemifield all patients consistently showed both an improvement of correct detections in the post-PAS block and a decline in the recovery block. The majority of patients, but not all, also showed a shift of the VF border toward the anopic hemifield in the post-PAS block, and in the recovery block the VF border was consistently shifted to the intact hemifield with reference to its position in the post-PAS block.

A two-factor (3×3) repeated measures ANOVA with measurement point in time (“Time”) and “Condition” as within-patient factors was conducted for correct detections of light flashes in the anopic hemifield (2–90° azimuth). The ANOVA revealed a significant main effect of the factor “Condition” (*F*[2,18] = 4.55, *p* = 0.025, *η*_p_^2^ = 0. 34) and a two-way interaction of “Time”×“Condition” (*F*[4,36] = 7.80, *p* = 0.00012, *η*_p_^2^ = 0.46), but no main effect of “Time” (*F*[2,18] = 2.65, *p* = 0.10, *η*_p_^2^ = 0.23), indicating an effect of the experimental condition that was specific to the measurement point in time (Figs. 6A, 7). Subsequent one-way ANOVAs for each hemifield condition with “Time” as factor showed a significant effect with PAS on the side of the anopic hemifield (*F*[2,18] = 9.87, *p* = 0.0013, *η*_p_^2^ = 0.52), but not for the remaining two conditions (*F*[2,18]≤1.64, *p*≥0.22, *η*_p_^2^≤0.15). Post-hoc comparisons, using paired *t*-tests, revealed that the mean percentage of correct detections obtained in the post-PAS block after PAS on the anopic side (9.56%, SE 2.01) was significantly higher than those of both pre-PAS (5.17%, SE 2.03; *t*[9] = 4.29, *p* = 0.0020) and recovery (5.09%, SE 1.30; *t*[9] = 4.09, *p* = 0.0027), while the recovery results did not differ from the pre-PAS data (*t*[9] = 0.07, *p* = 0.95). Thus, the percentage of correct visual detections in the post-PAS block was increased by 86.5% with reference to the mean of the pre-PAS and recovery measurements. Further subsequent one-way ANOVAs with “Condition” as factor showed a significant effect for the post-PAS measurement (*F*[2,18] = 12.89, *p* = 0.00034, *η*_p_^2^ = 0.59), but not for the remaining two measurement points in time (*F*[2,18]≤1.59, *p*≥0.23, *η*_p_^2^≤0.15). Post-hoc paired *t*-tests revealed that for the post-PAS block the percentage of correct detections after PAS on the anopic side (9.56%, SE 2.01) significantly differed from the control condition (4.95%, SE 1.81; *t*[9] = 6.63, *p* = 0.00010). The difference in the percentage of correct decisions between the intact-hemifield PAS (6.91%, SE 1.55) and the anopic-hemifield PAS only approached significance after Bonferroni correction (*t*[9] = 2.45, *p* = 0.037) and was not significant for the control condition (*t*[9] = 2.14, *p* = 0.061). Thus, with reference to the control condition, PAS on the side of the anopic hemifield increased the percentage of correct detections by 98.1%, while after PAS of the intact hemifield there was a numerical, non-significant trend for an enhancement that was about half that obtained after anopic-hemifield PAS and control condition.

For all conditions, significant changes in performance were absent in the intact hemifield, with even a slight numerical decrease in the percentage of correct detections after PAS in each case (mean difference pre- vs. post-PAS≤5.41%, SE 2.65; *t*[9]≤2.04, *p*≥0.071).

### Effect of PAS on visual field border

A two-factor (3×3) repeated measures ANOVA with “Time” and “Condition” as factors was conducted for the normalized VFBs (Figs. 6B, 7).There was an obvious numerical trend for a shift of the VFB toward the anopic side immediately after PAS, even though the ANOVA did not indicate significance for the main effects or the interaction (all *F*≤2.90, *p*≥0.11, *η*_p_^2^≤0.24).

### Control experiment

An additional control experiment was conducted with a subgroup of four patients (LHA3, LHA4, LHA6, RHA3) after completion of the main experimental sessions. Patients were tested as in the main session with PAS of the anopic side, except that a different PAS protocol was used: Sound bursts (duration 400 ms; onset/offset time 200 ms; sound pressure level and spectral content as in the main experiment; see Materials and Methods) were presented at a mean rate of of 0.2 Hz, with 3 s jitter. Instead of simultaneous presentation of sound stimuli from two different sources, each sound burst was presented from one loudspeaker. Sound locations changed between presentations following a random scheme, with positions between 14° and 76° (steps of 2°) in the patient's anopic hemifield. This very-low frequency stimulation protocol was applied in order to get some hints whether the effect obtained in the main experiment (see above) was specific to the high-frequency PAS protocol used, or was a consequence of the lateral acoustic stimulation *per se*. One-way ANOVAs for these data with “Time” as factor showed a significant effect in the main experiment (*F*[2,6] = 7.35, *p* = 0.024, *η*_p_^2^ = 0.71), but not for the control condition (*F*[2,6]≤1.55, *p* = 0.29, *η*_p_^2^≤0.34), thus suggesting that the effect shown in the main experiment was critically dependent on the specific stimulation protocol used.

## Discussion

These results showed that one hour of PAS on the side of the blind, but not of the intact, hemifield of patients with HA induced an improvement in visual detections by almost 100% within 30 min after PAS. This enhancement in performance was reversible and was reduced to baseline 1.5 h later. Also, there was a non-significant trend of a shift of the VFB toward the blind hemifield after PAS (see Figs. 5, 7). However, in sum, these results left open the question whether the genuine effect of PAS consisted in an enhancement of residual vision extending over large parts of the anopic field (as would be expected with an intensification of blindsight functions [65]) or in a dislocation of the transition zone between blind and sighted fields [66], [67], since each of these patterns was observed in some individual patients, without any consistent trend (see Fig. 7). Thus, it remained unclear whether the improvement in visual detections resulted from an enhancement of blindsight or from an activation of residual vision due to partial damage to the primary pathway.

The results are compatible with the view that the PAS used induced some activation of the residual visual pathways, even though the data left open the question of precisely which structures of the still available (extrastriate or primary) “residual” pathways were involved. In particular, the multimodal regions of the SC, that was spared by lesions in HA patients, may be a candidate substrate of the effect of PAS on visual performance. As sound stimuli were spatially arranged such that they were approximately covering one hemifield, one may assume that auditory or multisensory neurons in the contralateral SC were activated during presentation and may have driven short-term processes of synaptic plasticity of pathways originating in the SC and of their non-lesioned target areas in extrastriate cortex, either via the pulvinar [22], [23] or via the lateral geniculate nucleus [14]. These processes, possibly relying on a mechanism of LTP-like facilitation at the synaptic level, may have resulted in a reversible recruitment of the residual visual processing resources in HA, thus significantly enhancing visual sensitivity for a short time. In this respect, the central role of the multisensory circuits of the SC seems plausible, as the visual task used in this study was comparatively simple, merely requiring detection of light flashes appearing in darkness at random points in time [9]. The result that an effect of hemispatial PAS was only present on the anopic side (not on the intact side) can be explained by assuming that spatial auditory processing in the colliculo-cortical pathways of both cerebral hemispheres is – despite the extensive bilateral projections of the auditory system in general – contralaterally organized. On the one hand, PAS on the anopic side may have induced a selective activation of the intact colliculo-cortical pathway in the damaged hemisphere. On the other hand, PAS on the side of the intact field (ipsilateral to the lesion) may have had no significant influence on processing in the residual pathway of the damaged hemisphere. This finding is compatible with data from several mammalian species indicating the existence of a neural map of the contralateral auditory (multisensory) hemispace in the SC (for review, see [1]). In this context, the possibility has to be taken into account that our PAS paradigm, using synchronous presentation of *two* spatially disparate sound sources (which was adopted from previous non-auditory research), may not necessarily be essential for the result obtained here. Future studies may have to clarify whether beneficial effects of PAS can be elicited when only one single sound source is used.

In conclusion, it seems possible that PAS induced an intensification of multisensory features of “residual” structures, such as the SC. At the level of synaptic transmission, similar Hebbian mechanisms may be relevant for these cross-modal processes of plasticity as have been proposed by Dinse et al. [48] for unattended activation-based learning in the tactile modality. The repetitive (LTP-like) auditory stimulation on the side of the blind field may have induced synchronous neural activity in the circuits of the auditory and multisensory neurons in the contralateral SC (representing the affected hemispace) and its cortical target areas (possibly even those of the non-lesioned, ipsilateral hemisphere), thus modifying synaptic efficacy within the colliculo-cortical pathways, which may have resulted in improved visual processing therein. In this respect, our findings are in accordance with the previous studies that have demonstrated that perceptual learning can be induced by the variation of input statistics alone, without invocation of attention or reinforcement, and even without awareness of stimuli (for review, see [68]).

Alternatively, these results can be interpreted in terms of changes in visual attention. PAS could have increased sustained lateralized covert attention, resulting in temporarily improved visual detection. This interpretation seems plausible as the multisensory brain structures of the residual visual pathways, in particular the SC, are well-known to be crucially involved in visual attentional functions [20], [21]. Thus, PAS on the side of the anopic hemifield could have engaged multisensory mechanisms that have intensified visual attentional functions of the residual pathway. This explanation might be compatible with previous studies that have shown that visual improvements in HA patients can be obtained immediately in an attentional cueing task [69] and after long-term training with an attenion cue [70]. In order to shed some light on this issue, a further control condition was conducted in which, instead of application of two sound positions, a single sound source was presented at randomly varying locations on the side of the anopic hemifield. This type of PAS may have been suited to draw the patient's attention to the hemifield of PAS, but without the specific (LTP-like) protocol used in the main experiment. These results did not indicate any consistent improvement in visual detection under these conditions of PAS, thus arguing in favour of a specific effect induced by the PAS protocol in the main experiment, rather than any more general change in spatial attention. In accordance with this view, we did not find improvements on the intact side after PAS on the same side, as would be expected in case of an intensification of spatial attention. Nevertheless, on the basis of these data there is no conclusive evidence with respect to the question of whether more basic sensory or higher-order attentional mechanisms were temporarily changed after the PAS.

Although our initial hypothesis was primarily based on auditory-visual cross-modal processes in the secondary (extrastriate) pathway, one can not completely rule out that some elements of the primary (geniculostriate) pathway to V1 have survived in the damaged hemisphere and have preserved some residual visual functions [15]–[17] that could have been intensified by the PAS. If one assumes any role of the residual parts of the primary pathway in the effect shown here, the results might also be interpreted in terms of the cross-modal and attentional mechanisms as discussed above for the involvement of the secondary pathway. In particular, there is sufficient evidence indicating multisensory convergence as well as cross-modal effects of attention in V1, which might be mediated by projections from auditory cortex, parietal lobe, and superior temporal cortex (e.g., [33]–[39]).

Furthermore, it is possible that PAS induced an increased bias in favour of reporting awareness of visual stimuli rather than a genuine perceptual improvement [8]. This problem remains unsolved as reliable data on the phenomenological quality of the visual percept are not available. However, an analysis of responses in the intact hemifield after PAS on the same side indicated a non-significant trend of a decrease in performance (probably due to fatigue), which rather argued against this possibility.

Finally, it is conceivable that PAS induced an increase in overt, rather than covert, attention. As the duration of the visual stimulus (50 ms) was well below the minimum saccadic reaction times for visual targets in healthy subjects (around 100 ms; [71], [72]), the initiation of visually-guided saccades prior to stimulus offset can be excluded. Even though patients consistently followed the instruction of fixation on the fixation target (except one patient excluded from the study), it might be that PAS induced a bias in the frequency or amplitude of spontaneous self-paced saccades and/or eccentric fixation towards the stimulated side, such that flashes presented on this side necessarily felt more frequently in the intact visual field. For technical reasons, namely the optimization of our set-up for PAS, it was impossible to implement fundus controlled presentation of visual stimuli, which would have completely excluded this possibility. In this context, it has to be emphasized that the criticism of previous studies employing visual restitution training (see below) may not directly apply to the present study. It has been doubted whether the described improvements after such training were real or were based on adaptive oculomotor strategies, such as those mentioned above, which the patients developed during the training phase. But if such adaptive strategies would have played any role in our experiments, performance may have been continuously increasing over time, unlike the reversible improvement after PAS found here. Moreover, it is hard to imagine that the patients were aware of our hypotheses and changed their oculomotor behaviour accordingly between experimental blocks. Patients were naïve with respect to the exact scientific background of the experiment and our concrete expectations. However, as the general purpose of the study might be obvious by the procedure *per se*, and patients who had completed the first session might have obtained some further knowledge in this respect, the sequence of conditions was not balanced, but the most critical condition (blind-field PAS) was always conducted first. Notwithstanding, there was no increase in performance over sessions, as would be expected if improvements were based on explicit learning. In future studies it will, nevertheless, be necessary to employ fundus-controlled microperimetric methods, optimally using a scanning laser ophthalmoscope, to receive further insights into the effects of PAS on blind-field vision.

On the one hand, these data do not allow any conclusion about long-term effects of PAS. It was shown that the visual improvement had disappeared as soon as 1.5 h after one-time treatment. On the other hand, it is known from previous studies that have used passive stimulation for improving sensorimotor performance in subacute and chronic stroke patients that daily application for several weeks can induce long-lasting therapeutic effects [49]. Thus, it seems likely that repetition of PAS to HA patients induces longer-lasting or even permanent improvements of blind-field vision. The intriguing finding that already one-time application for one hour can induce improvements by about 100% is quite promising in this respect, but the therapeutic value of this treatment has still to be established. In particular, it has to be emphasized that the present data did neither demonstrate nor exclude a genuine *restitution* of parts of the blind field, which would bear the potential for improvement of more complex visual abilities, such as reading or spatial orientation.

The present study was based on a conception fundamentally different from previous approaches to change homonymous visual field defects by visual restitution training (e.g., [43], [69], [70], [65]–[67], [73]–[81]; for review, see [17]). Most importantly, it was shown that substantial improvements of blind-field vision can be also induced without requiring any active task nor explicit attention from the patient. If it should emerge in future studies that long-lasting improvement can be induced, passive stimulation may turn out to be an effective therapeutic alternative, with potentially higher compliance of patients to application than exhausting training procedures. Similarly important in this respect is the demonstration of cross-modal effects of passive stimulation. Compared with visual abilities, spatial hearing has been shown to be unusually robust to unilateral cortical lesions, most likely because of the generally bilateral organization of the auditory system, with a relatively weakly pronounced contralaterality in cortical processing: even after hemispherectomy, sound localization performance can be approximately normal [82], [83]. Cross-modal passive stimulation using sound stimuli thus could open the possibility for therapeutic intervention even in the case that very severe impairment of visual abilities limits the effectiveness of visual stimuli.

## Supporting Information

Trial Protocol S1 (PDF)
