# Supplementary material for: Passive Auditory Stimulation Improves Vision in Hemianopia
Source: PLoS One. 2012 May 29;7(5):e31603. doi: 10.1371/journal.pone.0031603 (PMC3362608; doi:10.1371/journal.pone.0031603)
Supplement: Trial Protocol S1 — (PDF) [file pone.0031603.s001.pdf]

ANTRAG

zur Beratung in ethischen und rechtlichen Fragen eines medizinischen Forschungsvorhabens am Menschen

I. Daten zum Projekt

1.

**Ort und Datum der Antragstellung**:
Bochum und Dortmund, 25.05.2005

2.

**Titel des Projektes** *(entsprechend dem deutschen Titel der Patienten-Information)*
Kortikale Integration multimodaler Objekt- und Rauminformation: Untersuchungen mit transkranieller Magnetstimulation (TMS), funktioneller Magnetresonanztomographie (fMRT), Elektroenzephalographie (EEG) und ereigniskorrelierten Potentialen (EKP) bei gesunden Probanden und psychophysische Untersuchungen von Patienten mit Hirnläsionen, M. Parkinson oder M. Huntington.

3.

**Name u. Anschrift der/des *hiesigen* verantwortlichen Projektleiterin/Projektleiters**	
PD Dr. rer. nat. Jörg Lewald<br><br>Institut für Arbeitsphysiologie an der Universität Dortmund<br>Projektgruppe Kognitive Neurophysiologie<br>Ardeystr. 67<br>44139 Dortmund<br><br>und<br><br>Fakultät für Psychologie<br>AE Kognitions- und Umweltpsychologie<br>Ruhr-Universität Bochum<br>44780 Bochum	
Bitte für Rückfragen angeben:	
Telefon-Nummer:	302 22074 oder 0231/1084 263
Fax-Nummer:	302 02074
E-Mail:	joerg.lewald@rub.de

3. A

**Bei Multicenter-Studien, koordinierendes Zentrum der Studie**:
entfällt

3. B

**Name und Anschrift *Leiter/in der Klinischen Prüfung*:**	
eine klinische Prüfung ist nicht Gegenstand der Studie	
Bitte für Rückfragen angeben:	
Telefon-Nummer:	
Fax-Nummer:	
E-Mail:	

3. C Liegen Voten anderer Ethik-Kommissionen vor? Wenn ja, welche?

Ein auf Antrag vom 11.12.2001 erteiltes positives Votum der Ethik-Kommission der Medizinischen Fakultät der Ruhr-Universität Bochum vom 23.01.2002 (Reg.-Nr. 1800) zu dem laufenden Projekt liegt vor. Der vorliegende Antrag ist in wesentlichen Teilen mit dem begutachteten Antrag vom 11.12.2001 identisch. Ergänzungen des vorliegenden Antrags gegenüber dem Antrag vom 11.12.2001 wurden in unserem Amendment vom 21.02.2005 ausführlich erläutert, welches von der Kommission nicht bearbeitet wurde. Die Unterlagen sind in Kopie beigefügt.

4. **Weitere Abteilungen/Institute (mit Untersuchern), die an der Studie beteiligt sind**:

A	Institut für Arbeitsphysiologie an der Universität Dortmund<br>Projektgruppe Kognitive Neurophysiologie<br>PD Dr. med. Michael Falkenstein<br>Ardeystr. 67<br>44139 Dortmund
B	Kliniken Dortmund<br>Neurologische Klinik<br>Prof. Dr. med. Michael Schwarz<br>Beurhausstr. 40<br>44137 Dortmund
C	Ruhr-Universität Bochum<br>Fakultät für Psychologie<br>AE Kognitions- und Umweltpsychologie<br>Dr. Stephan Getzmann<br>Universitätsstr. 142<br>44799 Bochum
D	Universität Witten/Herdecke<br>Institut für Mikrotherapie<br>Dr. Martin Busch<br>Universitätsstr. 142<br>44799 Bochum

5. **Sponsor**

Name:	entfällt
Adresse:	
Kontaktperson:	
Telefon:	
Fax	
e-mail:	

6. **Rechnungsanschrift für Gebühren der Ethik-Kommission** :
Bitte vollständige Anschrift <u>*mit*</u> *Ansprechpartner angeben.*

Firma/Klinik:	Der Erlaß der Gebühren gem §6 Abs. 2 der Satzung der Ethikkommissionen wird beantragt, da die Förderung durch die DFG erfolgt (Az. FA 211/17-1)
Abteilung:	
Ansprechpartner:	
Straße:	
PLZ/Ort:	

II. Forschungsvorhaben

1.

**<u>Kurzer</u> Abriss des Studien-Projektes** *(ca. 20 Zeilen)*:
Die Frage nach den neuralen Korrelaten der Verarbeitung auditiver Rauminformation in kortikalen Gehirnarealen außerhalb des primären auditorischen Kortex ist bisher weitgehend ungeklärt. Auch über die kortikalen Prozesse, die zu einer Integration auditiver und visueller Raum- und Objektinformation führen, ist noch wenig bekannt. Diese beiden Fragestellungen werden mit der bildgeführten repetitiven transkraniellen kortikalen Magnetstimulation (rTMS) angegangen, wobei die Bildführung mittels zuvor erstellter magnetresonanztomographischer Darstellung (MRT) des Cortex erfolgt. Im Vordergrund stehen hierbei die Effekte der Magnetstimulation verschiedener Kortexareale auf die Schallokalisation bei sehenden und blinden Probanden. Ferner werden psychophysische Experimente zum räumlichen Hören bei Patienten mit spezifischen Hirnläsionen oder Störungen der Basalganglien sowie blinden Probanden durchgeführt. Schließlich werden mögliche zentralnervöse Korrelate der auditiven Lokalisation mit elektrophysiologischen (Elektroenzephalographie, EEG; ereigniskorrelierte Potentiale, EKP) und neuroradiologischen Methoden (funktionelle Magnetresonanztomographie, fMRT) bei gesunden sehenden und bei blinden Probanden untersucht. Auf der Grundlage dieser Untersuchungen sollen dann im weiteren Verlauf des Projektes relativ einfache auditiv-visuelle Reizkombinationen bei normalen Probanden und Patienten mit Hirnläsionen eingesetzt werden, die die Anforderungen an die Merkmalsanalyse im wesentlichen auf die Integration von Raumkoordinaten und Zeitstrukturen der auditiven und visuellen Reize reduzieren. Das Ziel ist eine möglichst genaue Charakterisierung der spezifischen Funktionen einzelner Hirnareale bei diesen Integrationsprozessen, wie sie bei bisherigen Untersuchungen mit bildgebenden Verfahren nicht möglich war. Letztlich erwarten wir uns Aufschlüsse über das noch ungelöste Problem der Bildung kohärenter kognitiver Repräsentationen von Objekten im Raum, die die perzeptive Integration und schnelle Analyse einer Vielzahl von Objektmerkmalen erfordert.

2. **<u>Ausführliches</u> Studienprotokoll/Produktbeschreibung u. Investigator's Brochure bitte als Anlage beifügen**:

(bitte ankreuzen):

	Studien-Protokoll	Version/ Datum:<br>Eine Arzneimittelprüfung ist nicht Gegenstand der Studie. Eine ausführliche Darstellung des experimentellen Ansatzes findet sich im Antrag vom 11.12.2001 (in der Anlage).
	eventuell Folge-Protokoll	Version/ Datum:
	Investigator's Brochure/Fachinformation:	Version/ Datum:

3. **Der Antrag bezieht sich auf**

(bitte ankreuzen):

Arzneimittelgesetz		
	Phase 1	
	Phase 2	
	Phase 3	
	Phase 4	
Strahlenschutzverordnung		
Röntgenverordnung		
Medizinproduktegesetz		
Transfusionsgesetz		
Gentechnikgesetz		
keines der genannten Gesetze		**X**

4. **Angaben zum Medizinprodukt** *(Betrifft nur Studien gemäß MPG)*

**Bezeichnung des Produktes**:				
entfällt				
**Hersteller**				
Zugelassen für die Prüfindikation	ja		nein	
Zugelassen, aber für eine andere Indikation:	ja		nein	
Das Medizinprodukt trägt ein CE-Zeichen:	ja		nein	
Die Produktbroschüre liegt bei:	ja		nein	

5. **Anliegen/Rationale des Forschungsvorhabens**

(Zutreffendes bitte ankreuzen):

Substanzprüfung		Therapieoptimierung	
Neue Indikationen		Neue Behandlungsverfahren	
Diagnose-oder Indikationsprüfung		Vergleich mehrerer Verfahren	
Erprobung/Entwicklung med. Geräte		Gewebeanalyse	
Gentechnische Mittelproduktion/Therapie		Med.-wiss. Daten	
Epidemiologie		Grundlagenforschung	**X**

6.

**Zielkriterien: (Primary/secondary endpoints)**:
entfällt

7. **Merkmale der Studie**
(Zutreffendes bitte ankreuzen)

Monozentrisch		multizentrisch	
Verblindet		offen	
Randomisiert		vergleichend	
Kontrolle gegen Standardtherapie		Plazebokontrolle	
Retrospektiv		prospektiv	
Crossover		Pilotstudie	

Eine Arzneimittelprüfung ist nicht Gegenstand der Studie.

III. Fragen zu den Patienten und Probanden:

1.

1.	**Wie viele Patienten/Probanden sollen in die Studie aufgenommen werden?**				
	**a) Untersuchungen mit rTMS, MRT, fMRT und EEG/EKP bei gesunden Probanden:**<br><br>voraussichtlich 80<br><br>**b) Psychophysische Untersuchungen bei Patienten:**<br><br>Für die geplanten Patienten-Studien werden Patienten untersucht, die in die Städtischen Kliniken Dortmund eingeliefert werden. Im Alter entsprechende Personen ohne neurologische Störungen sollen als Kontrollprobanden gewonnen werden. Die Gesamtzahl beider Gruppen wird voraussichtlich 80 betragen.				
2.	**Studienplanung. Eine Fallzahlenberechnung wurde durchgeführt**.<br>*(Fallzahlenberechnung mit Kennzahlen (Fehler 1. und 2. Art, z.B. MW, Standardabweichung)*:				
	Eine Fallzahlenberechnung entfällt. Erfahrungsgemäß ist bei allen verwendeten Methoden für jede einzelne Fragestellung eine Anzahl von 12-15 Probanden ausreichend. Im Falle der Untersuchung von Patienten kommt eine gleich große Anzahl an gesunden Kontrollprobanden hinzu.				
3.	**Erwartete Anzahl von Studienabbrechern**:				
	Nach bisherigen Erfahrungen sind keine oder nur sehr wenige Studienabbrecher zu erwarten.				
4.	**Geplante statistische Analyse**				
	Intention-to-treat	ja		nein	X
	Per protocol	ja		nein	X
	Zwischenauswertung	ja		nein	X
5.	**Abbruchkriterien**				
	Jedes Experiment kann seitens des Probanden jederzeit abgebrochen werden.				

6.	**Geplante statistische Verfahren**:
	Die statistische Analyse der anonymisierten Daten erfolgt mittels Varianzanalyse bzw. t-Tests unter Verwendung des Programmpaketes SPSS for Windows.Bei Untersuchungen mit rTMS erfolgt ein statistischer Vergleich der Leistung vor und nach rTMS (repeated measures design). Als Kontrolle dient Plazebo-rTMS (mittels einer speziellen Plazebospule).<br><br>Bei psychophysischen Untersuchungen mit Patienten erfolgt ein Vergleich von Patienten mit entsprechend dem Alter und dem Geschlecht gematchten gesunden Kontrollprobanden.
7.	**Bei multizentrischen Studien, wie viele Zentren nehmen teil**?
	entfällt
8.	**Wie lange ist der Rekrutierungszeitraum?**
	3 Jahre
9.	**Einschlusskriterien**:
	**a) Untersuchungen mit rTMS, MRT, fMRT und EEG/EKP bei gesunden Probanden:**<br>Als Probanden sollen in der Regel Studierende der Universitäten Bochum und Dortmund gewonnen werden. Darüber hinaus sollen kongenital blinde sowie spät erblindete gesunde Probanden einbezogen werden. Weitere Kriterien für die Auswahl sind: Alter 18-45 Jahre und normale Hörfähigkeit.<br><br>**b) Psychophysische Untersuchungen bei Patienten:**<br>Für die geplante Patienten-Studie werden Patienten mit umschriebenen Hirnschädigungen des frontalen, okzipitalen oder temporo-parietalen Cortex oder der Basalganglien nach Infarkt oder Blutung oder Patienten mit M. Parkinson oder M. Huntington untersucht, die in die Städtischen Kliniken Dortmund eingeliefert werden. Im Alter entsprechende Personen ohne neurologische Störungen sollen als Kontrollprobanden gewonnen werden. Die Patienten müssen körperlich und intellektuell in der Lage sein, konzentriert an Experimenten teilzunehmen. Außerdem muß eine altersmäßig normale Hörfähigkeit vorhanden sein.

10.	**Ausschlusskriterien**:			
	**a) Untersuchungen mit rTMS, MRT, fMRT und EEG/EKP bei gesunden Probanden:**<br>Es dürfen keinerlei körperliche oder geistige Behinderungen sowie insbesondere keine neurologischen Störungen vorliegen. Absolute Ausschlußkriterien sind: Metallimplantate (Cochlea-Implantate, Gefäßclips oder andere cerebrale Metallimplantate), cerebrale Anfallsanamnese, Erkrankungen des Herzens, Herzschrittmacher, Schwangerschaft, Medikation mit Psychopharmaka, Epilepsie bzw. bekannte Fälle von Epilepsie in der Familie.<br><br>**b) Psychophysische Untersuchungen bei Patienten:**<br>Ausschlußkriterien sind Schwerhörigkeit, Aphasie und Demenz.			
11.	**Besteht eine Versicherung**?			*Bitte ankreuzen:*
	ja			
	nein	X		
	**Bitte Versicherungsgesellschaft angeben**:			*Kopie der Versicherungspolice beifügen*
12.	**Entgelt oder sonstige Vergünstigungen für Versuchspersonen**			
	ja	X	€ 7,50/h nach Aufwand (i.d.R. € 15-30 pro Sitzung); bei Studierenden der Psychologie auch Anrechnung von Pflicht-Versuchspersonenstunden	*Bitte ankreuzen:*
	nein			

2. **Welche Maßnahmen bzw. Behandlungen werden ausschließlich studienbezogen durchgeführt?**

Bitte führen Sie die Art der studienbezogenen Maßnahmen an, sowie die Anzahl und den Zeitraum. Z. B. Blutabnahem, 5 x, 3 Monate, 200 ml

Art:	Anzahl/Dosis:	Zeitraum:	insgesamt:
rTMS	pro Versuchstag 2 x 900 Pulse bei 1 Hz (je 15 min, dazwischen mind. 20 min Pause) und einer Intensität von 60%	max. 8 Versuchstage in 3 Monaten	max. 14.400 Pulse
MRT	1 Untersuchung, (ca. 20 min)	jeweils vor Beginn der rTMS-Experimente	einmalig
fMRT	1 Untersuchung (ca. 40 min)	einmalig	einmalig
Psychophysische Messungen	ca. 1-2 h Dauer einschl. mehrerer Pausen	3 Jahre	max. etwa 10 Untersuchungen

IV. Risiko-Nutzen Abwägung

**In diesem Versuch besteht <u>folgender Nutzen</u> für den Patienten bzw. Probanden**:
Mögliche Aufklärung von bisher nicht diagnostizierten Erkrankungen.
**In diesem Versuch bestehen <u>folgende Risiken</u> für den Patienten bzw. Probanden**:
Bei allen eingesetzten Methoden handelt es sich um nicht-invasive, schmerzfreie und sehr risikoarme Methoden, die zum diagnostischen Repertoire einer neurologischen Klinik gehören.<br><br>Bezüglich der transkraniellen Magnetstimulation sei auf die folgende ausführliche Übersicht über die möglichen Risiken verwiesen: Wassermann EM Risk and safety of repetitive transcranial magnetic stimulation: report and suggested guidelines from the International Workshop on the Safety of Repetitive Transcranial Magnetic Stimulation, June 5-7, 1996. Electroencephalogr Clin Neurophysiol 108: 1-16 (1998). Die dort genannten Grenzwerte der Stimulationsfrequenz und -amplitude werden in den geplanten Experimenten bei weitem unterschritten. In einer Reihe von Arbeiten ist bisher untersucht worden, ob die transkranielle Magnetstimulation zeitweise oder bleibende Nebenwirkungen verursacht. Die Entladung der Magnetspule ist mit einem Klickgeräusch verbunden, dessen Lautstärke bis zu 100 dB SPL erreicht. Beeinträchtigungen des Hörvermögens sind bei den geplanten Untersuchungen jedoch ausgeschlossen, da die Stimulation unter Verwendung eines speziellen Lärmschutzes mit einer Abschwächung von >40 dB erfolgt. Weiterhin ist bei einigen Probanden ein vorübergehender Kopfschmerz beschrieben, der gut auf Analgetika wie Acetysalicylsäure oder Paracetamol anspricht. Es ist außerdem eine Aktivierung von bestehenden epileptischen Foci durch transkranielle Magnetstimulation beschrieben. Eine positive Anfallsanamnese sowie das Vorliegen von Epilepsie in der Familienanamnese stellen deshalb eine Kontraindikation für den Einsatz von transkranieller Magnetstimulation dar, entsprechende Personen werden aus dieser Untersuchung ausgeschlossen. Dem extrem niedrigen Anfallsrisiko wird weiterhin dadurch begegnet, daß der Untersuchungsraum mit einem Notfallkoffer ausgestattet ist und eine unmittelbare Erreichbarkeit eines Arztes bzw. einer notfallmedizinisch ausgebildeten Person während der Untersuchung gewährleistet ist.
**Zu erwartender <u>Nutzen für die Medizinische Wissenschaft</u>**?
Wir erwarten Aufschlüsse über die bisher unzureichend beantwortete Frage nach den neuralen Korrelaten der Schallokalisation sowie der Integration auditiver und visueller Raum- und Objektinformation im Cortex sowie den Basalganglien (s. Abschnitt II.1.).

entfällt

....	....
Stempel/Unterschrift<br>Ärztlicher Direktor der Klinik/ des Instituts	Stempel/Unterschrift<br>Projektleiter

Anlagen:
*(zutreffendes bitte ankreuzen, *: obligate Anlage)*

* Studienprotokoll	s. Antrag vom 11.12.2001 (Anlage)
* Patientenaufklärung	**X**
Kopie Versicherungspolice	
vorliegende Ethikvoten	**X**
begutachteter Antrag vom 11.12.2001	**X**
Amendment vom 21.02.2005	**X**
